# The genome sequence of the Cow Parsley Leaf Beetle, *Chrysolina oricalcia* (O.F. Müller, 1776)

**DOI:** 10.12688/wellcomeopenres.19985.1

**Published:** 2023-09-11

**Authors:** Olga Sivell, Duncan Sivell, Michael Geiser, Natural History Museum Genome Acquisition Lab, Darwin Tree of Life Barcoding collective, Wellcome Sanger Institute Tree of Life programme, Wellcome Sanger Institute Scientific Operations: DNA Pipelines collective, Tree of Life Core Informatics collective, Darwin Tree of Life Consortium

**Affiliations:** 1 Natural History Museum, London, England, UK

**Keywords:** Chrysolina oricalcia, Cow Parsley Leaf Beetle, genome sequence, chromosomal, Coleoptera

## Abstract

We present a genome assembly from an individual Cow Parsley Leaf Beetle *Chrysolina oricalcia* (the Cow Parsley Leaf Beetle; Arthropoda; Insecta; Coleoptera; Chrysomelidae). The genome sequence is 1,423.4 megabases in span. Most of the assembly is scaffolded into 22 chromosomal pseudomolecules, including the X sex chromosome. The mitochondrial genome has also been assembled and is 16.93 kilobases in length. Gene annotation of this assembly on Ensembl identified 35,990 protein coding genes.

## Species taxonomy

Eukaryota; Metazoa; Ecdysozoa; Arthropoda; Hexapoda; Insecta; Pterygota; Neoptera; Endopterygota; Coleoptera; Polyphaga; Cucujiformia; Chrysomeloidea; Chrysomelidae; Chrysomelinae; Doryphorini; Chrysolinina; *Chrysolina*; subgenus *Sulcicollis*; *Chrysolina oricalcia* (O.F. Müller, 1776) (NCBI:txid1587174).

## Background

Chrysomelidae (leaf beetles) are one of the most diverse clades within Coleoptera, currently containing over 40,000 species, of which about 250 are recorded from the British Isles ( Duff, 2018). The genus *Chrysolina* (Motschulsky, 1860) is one of the most speciose genera within the subfamily Chrysomelinae, with almost 500 species currently considered valid ( Bieńkowski, 2007; Kippenberg, 2010). Nineteen *Chrysolina* species occur on the British Isles ( Duff, 2018). According to Kippenberg (2010), *Chrysolina* is classified in tribe Doryphorini, subtribe Chrysolinina. The five species classified in subgenus *Sulcicollis* (Sahlberg, 1913) are primarily distributed in the western Palaearctic region, with *C. chalcites* (Germar, 1824) and *C. oricalcia* (O.F. Müller, 1776) reaching as far as western Siberia and Mongolia ( Bieńkowski, 2007; Kippenberg, 2010).

*Chrysolina oricalcia*, the Cow Parsley Leaf Beetle, is a 6–9.5 mm long, unicolourous dark blue (sometimes greenish, purple, coppery, or almost black) leaf beetle. It can be distinguished from other British species of the genus by the very regular, coarse, but rather sparse puncture rows on the otherwise smooth and shining elytra in combination with the sharp and almost straight groove running parallel to the pronotal margins ( Duff, 2016). The latter character is a distinguishing feature of the subgenus *Sulcicollis*.

Members of Chrysomelidae are all phytophagous. While some species are polyphagous, most of them are strictly associated with certain families or species of higher plants (monophagous or oligophagous), both for their larval development and adult feeding. *Chrysolina oricalcia* is considered an oligophagous species, using various Apiaceae as its larval and adult host plants, but with a strong preference for Cow Parsley, *Anthriscus sylvestris* ( Rheinheimer & Hassler, 2018). Adults of *C. oricalcia* are mostly crepuscular or nocturnal. They hatch in late summer, but are found most frequently from April to June, after re-emerging from diapause. Females are ovoviviparous ( Bontems, 1985). Larvae are external leaf feeders on their host plants, where they can be encountered from April until late summer, before pupating in the soil ( Rheinheimer & Hassler, 2018). Cox and Broad (2020) reported parasitism of the larvae of *C. oricalcia* by an ichneumonid wasp *Nepiesta mandibularis* (Holmgren).

*Chrysolina oricalcia* is the only species of its subgenus occurring on the British Isles. Its overall distribution includes most of Europe excluding the Iberian Peninsula and the extreme North, as well as parts of Turkey and Mongolia. Up to the 1990s it was considered a scarce and potentially declining species in the UK, given the status “Notable B” in Hyman and Parsons (1992). It has however since increased in abundance and can now be considered a common species particularly in the South-East of England, East Anglia, and the West Midlands ( Cox, 2007; James, 2018), but only known from few localities in Scotland ( Ramsay, 2000). It was reassessed as “Least Concern” in the most recent conservation status review by Hubble (2014). The species is most often found in humid woodland ecotones, which can include parks and gardens, wherever large stands of *Anthriscus sylvestris* grow ( Rheinheimer & Hassler, 2018 and own observations in SE England).

**Figure 1.**
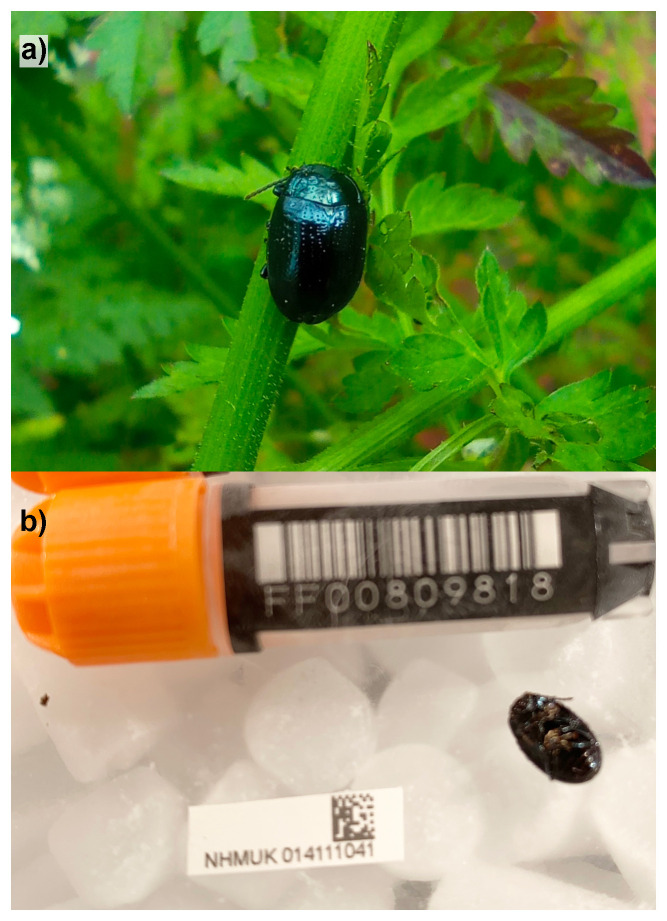
**a**) *Chrysolina oricalcia* photographed by Michael Geiser in Gunnersbury Park on the 2023-05-21 (not the specimen used for genome sequencing). **b**) Photograph of the *Chrysolina oricalcia* specimen (NHMUK014111041) used for genome sequencing.

The high-quality genome of *Chrysolina oricalcia* was sequenced from a single specimen (NHMUK014111041; SAMEA7520950) from Wigmore Park, Luton, UK ( Figure 1b)). It will aid research on taxonomy, phylogeny, and biology of this species. The genome was sequenced as part of the Darwin Tree of Life Project, a collaborative effort to sequence all named eukaryotic species in the Atlantic Archipelago of Britain and Ireland.

## Genome sequence report

The genome was sequenced from one male *Chrysolina oricalcia* ( Figure 1b)) collected from Wigmore Park, Luton (51.88, –0.37). A total of 45-fold coverage in Pacific Biosciences single-molecule HiFi long reads and 23-fold coverage in 10X Genomics read clouds were generated. Primary assembly contigs were scaffolded with chromosome conformation Hi-C data. Manual assembly curation corrected 92 missing joins or misjoins and removed two haplotypic duplications, reducing the scaffold number by 43.15%, and increasing the scaffold N50 by 99.91%.

**Figure 2.**
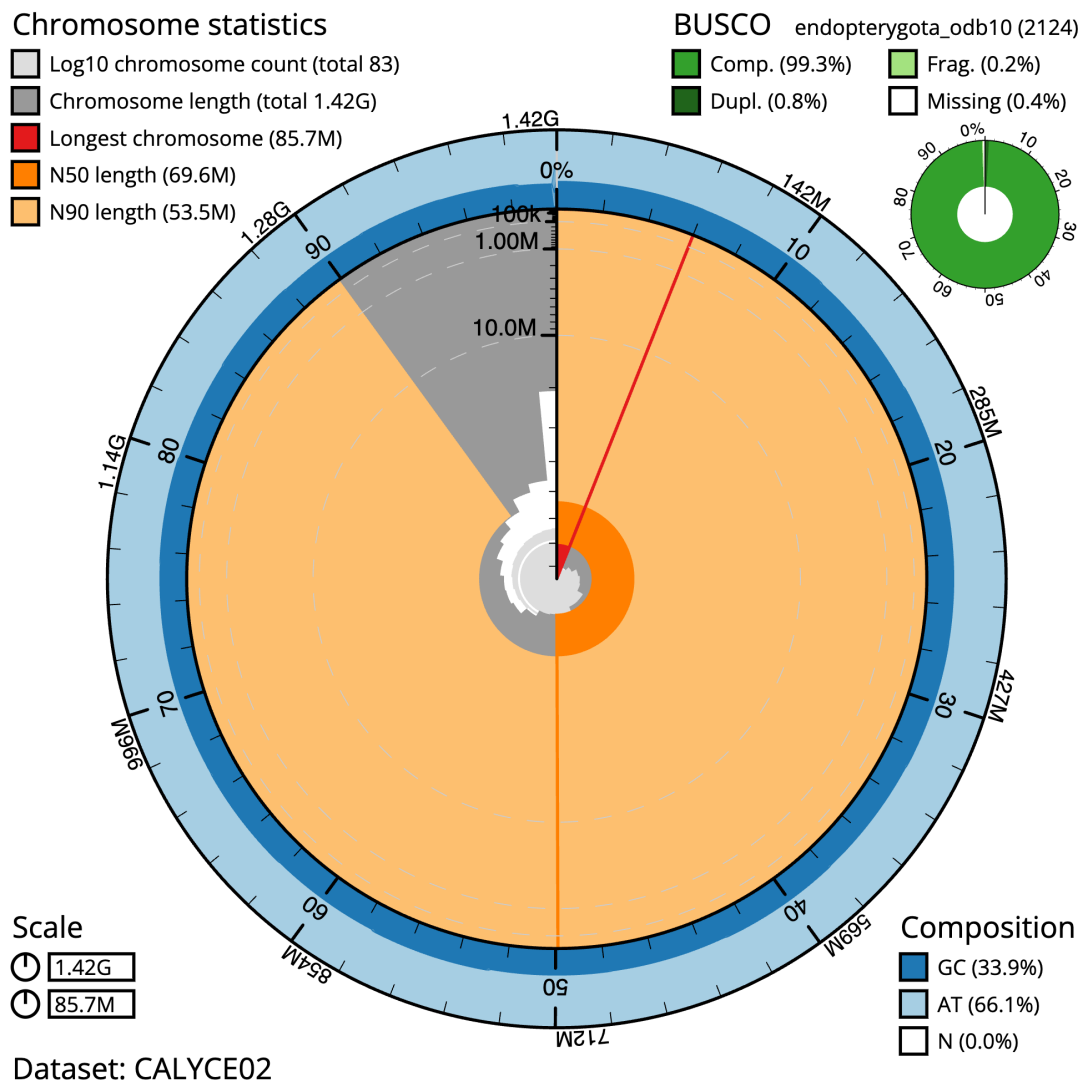
Genome assembly of *Chrysolina oricalcia*, icChrOric1.2: metrics. The BlobToolKit Snailplot shows N50 metrics and BUSCO gene completeness. The main plot is divided into 1,000 size-ordered bins around the circumference with each bin representing 0.1% of the 1,423,453,393 bp assembly. The distribution of scaffold lengths is shown in dark grey with the plot radius scaled to the longest scaffold present in the assembly (85,697,287 bp, shown in red). Orange and pale-orange arcs show the N50 and N90 scaffold lengths (69,622,592 and 53,506,149 bp), respectively. The pale grey spiral shows the cumulative scaffold count on a log scale with white scale lines showing successive orders of magnitude. The blue and pale-blue area around the outside of the plot shows the distribution of GC, AT and N percentages in the same bins as the inner plot. A summary of complete, fragmented, duplicated and missing BUSCO genes in the endopterygota_odb10 set is shown in the top right ..An interactive version of this figure is available at https://blobtoolkit.genomehubs.org/view/icChrOric1.2/dataset/CALYCE02/snail.

The final assembly has a total length of 1423.4 Mb in 82 sequence scaffolds with a scaffold N50 of 69.6 Mb ( Table 1). Most (99.84%) of the assembly sequence was assigned to 22 chromosomal-level scaffolds, representing 21 autosomes and the X sex chromosome. Chromosome-scale scaffolds confirmed by the Hi-C data are named in order of size ( Figure 2– Figure 5; Table 2). While not fully phased, the assembly deposited is of one haplotype. Contigs corresponding to the second haplotype have also been deposited. The mitochondrial genome was also assembled and can be found as a contig within the multifasta file of the genome submission.

**Table 1.** Genome data for *Chrysolina oricalcia*, icChrOric1.2.

Project accession data		
Assembly identifier	icChrOric1.2	
Species	*Chrysolina oricalcia*	
Specimen	icChrOric1	
NCBI taxonomy ID	1587174	
BioProject	PRJEB52653	
BioSample ID	SAMEA7520950	
Isolate information	icChrOric1, abdomen (DNA sequencing and Hi-C scaffolding)	
Assembly metrics *		*Benchmark*
Consensus quality (QV)	57.8	*≥ 50*
*k*-mer completeness	99.99%	*≥ 95%*
BUSCO **	C:99.3%[S:98.5%,D:0.8%], F:0.2%,M:0.4%,n:2,124	*C ≥ 95%*
Percentage of assembly mapped to chromosomes	99.84%	*≥ 95%*
Sex chromosomes	X chromosome	*localised homologous* *pairs*
Organelles	Mitochondrial genome assembled	*complete single alleles*
Raw data accessions		
PacificBiosciences SEQUEL II	ERR9709340, ERR9709339, ERR9709338	
10X Genomics Illumina	ERR9710917, ERR9710919, ERR9710918, ERR9710920	
Hi-C Illumina	ERR9710921	
Genome assembly		
Assembly accession	GCA_944452925.2	
*Accession of alternate haplotype*	GCA_944452915.2	
Span (Mb)	1,423.4	
Number of contigs	506	
Contig N50 length (Mb)	6.3	
Number of scaffolds	82	
Scaffold N50 length (Mb)	69.6	
Longest scaffold (Mb)	85.7	
Genome annotation		
Number of protein-coding genes	35,990	
Number of gene transcripts	36,271	

* Assembly metric benchmarks are adapted from column VGP-2020 of “Table 1: Proposed standards and metrics for defining genome assembly quality” from ( Rhie *et al.*, 2021). ** BUSCO scores based on the endopterygota_odb10 BUSCO set using v5.3.2. C = complete [S = single copy, D = duplicated], F = fragmented, M = missing, n = number of orthologues in comparison. A full set of BUSCO scores is available at https://blobtoolkit.genomehubs.org/view/icChrOric1.2/dataset/CALYCE02/busco.

**Figure 3.**
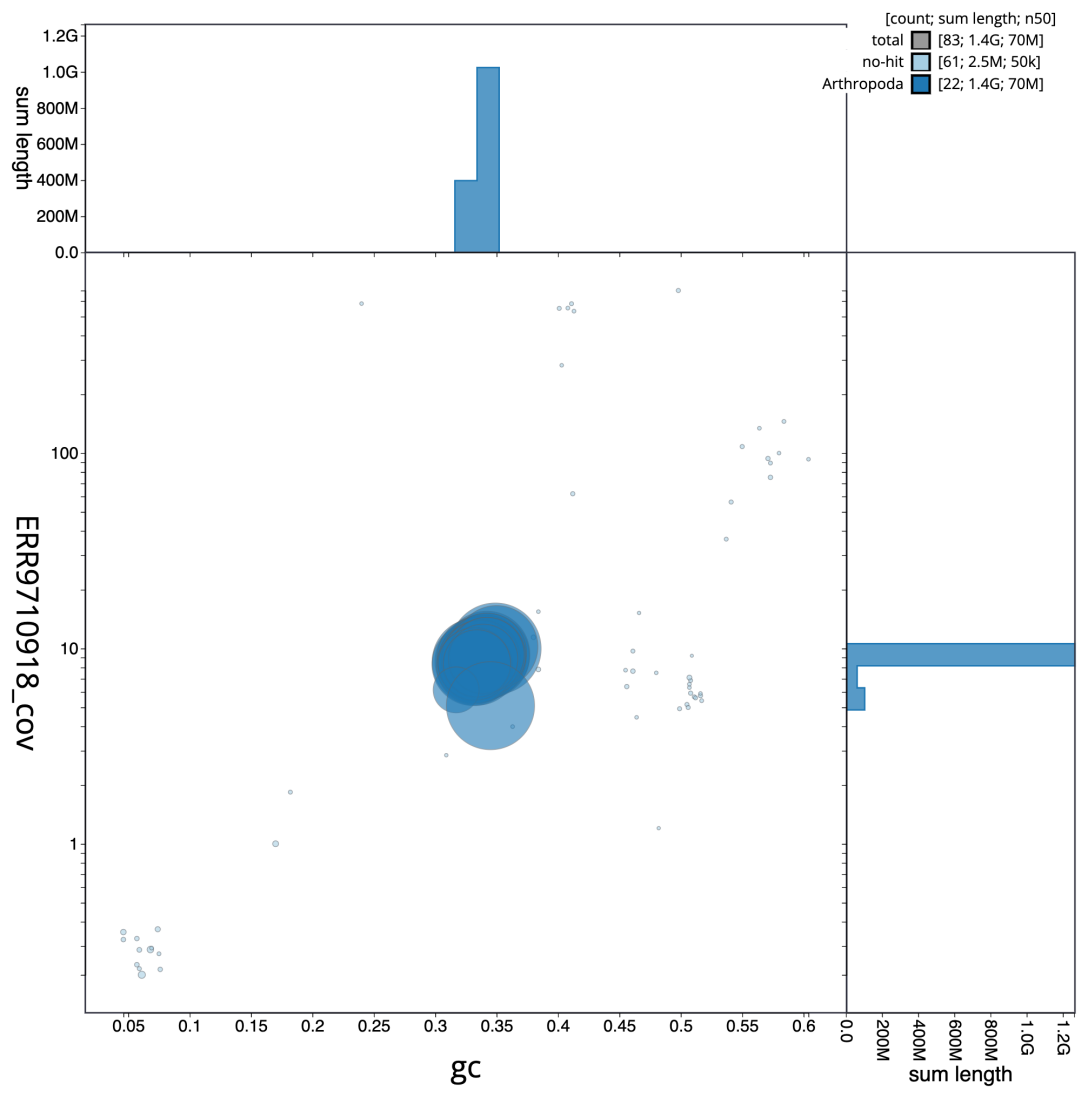
Genome assembly of *Chrysolina oricalcia*, icChrOric1.2: BlobToolKit GC-coverage plot. Scaffolds are coloured by phylum. Circles are sized in proportion to scaffold length. Histograms show the distribution of scaffold length sum along each axis. An interactive version of this figure is available at https://blobtoolkit.genomehubs.org/view/icChrOric1.2/dataset/CALYCE02/blob.

**Figure 4.**
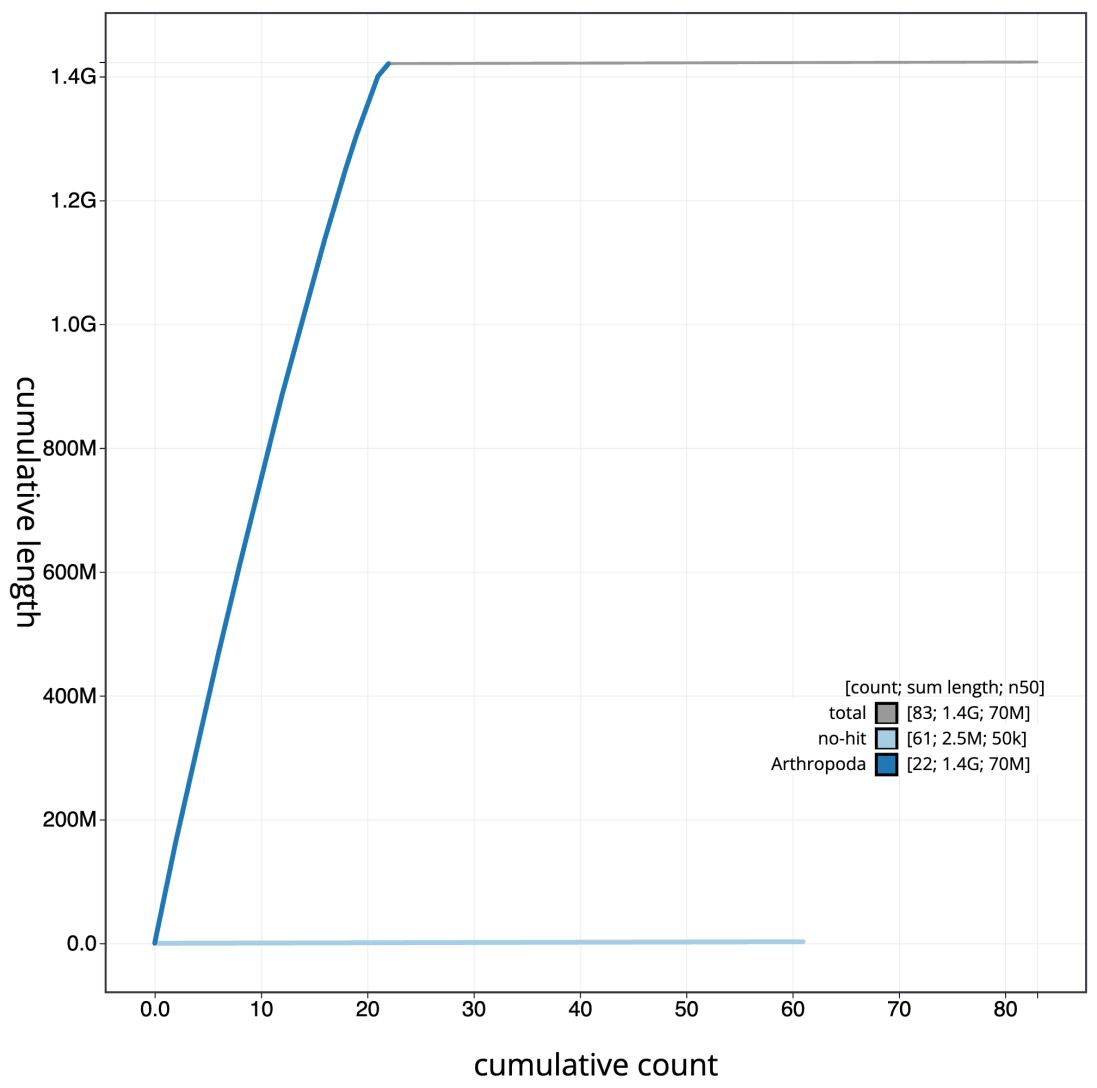
Genome assembly of *Chrysolina oricalcia*, icChrOric1.2: BlobToolKit cumulative sequence plot. The grey line shows cumulative length for all scaffolds. Coloured lines show cumulative lengths of scaffolds assigned to each phylum using the buscogenes taxrule. An interactive version of this figure is available at https://blobtoolkit.genomehubs.org/view/icChrOric1.2/dataset/CALYCE02/cumulative.

**Figure 5.**
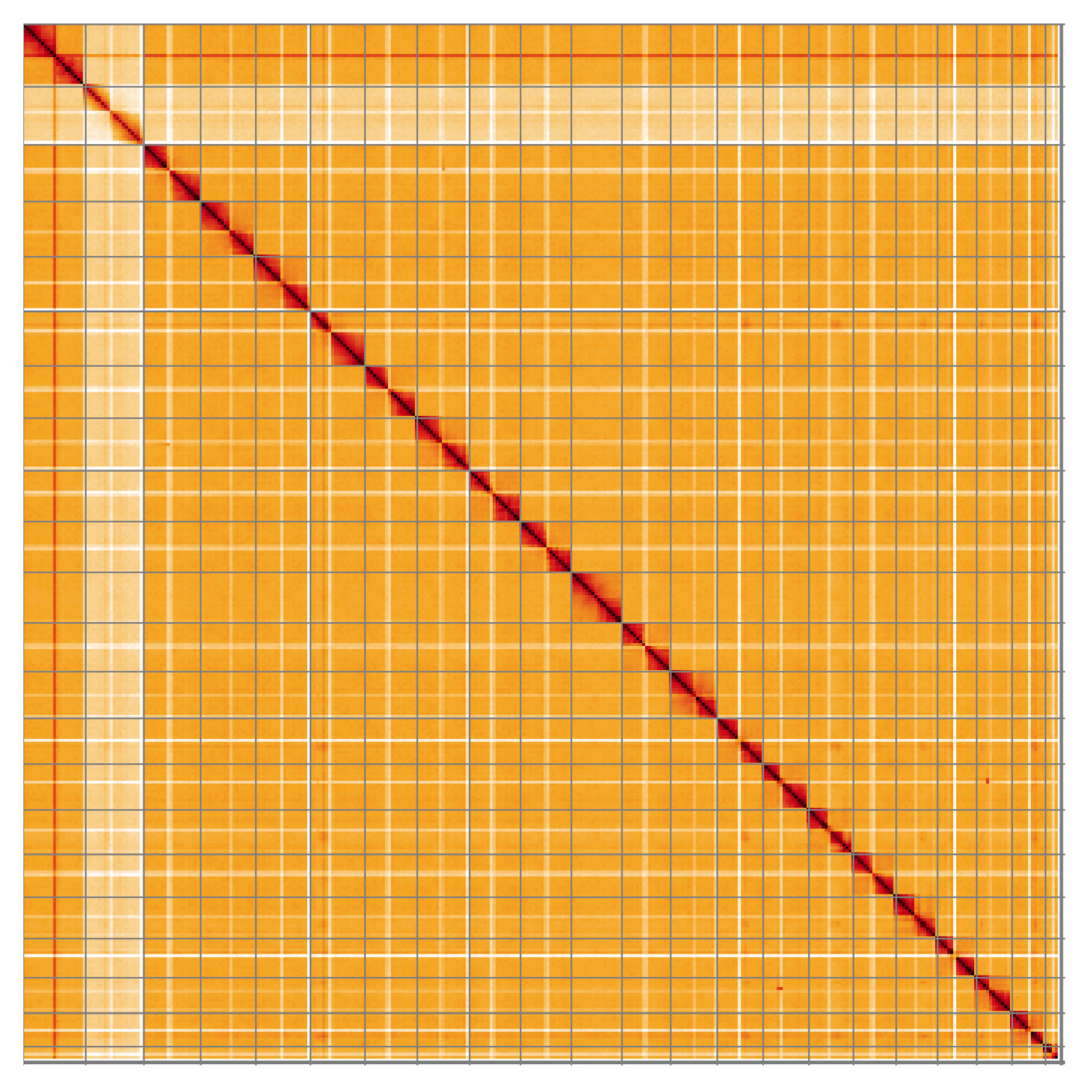
Genome assembly of *Chrysolina oricalcia*, icChrOric1.2: Hi-C contact map of the icChrOric1.2 assembly, visualised using HiGlass. Chromosomes are shown in order of size from left to right and top to bottom. An interactive version of this figure may be viewed at https://genome-note-higlass.tol.sanger.ac.uk/l/?d=I92pMEcZQxuyGSgz8qBnKQ.

**Table 2.** Chromosomal pseudomolecules in the genome assembly of *Chrysolina oricalcia*, icChrOric1.

INSDC accession	Chromosome	Length (Mb)	GC%
OX101808.2	1	85.7	35.0
OX101810.2	2	77.76	33.5
OX101811.2	3	75.56	34.0
OX101812.2	4	74.89	34.0
OX101813.2	5	74.57	34.0
OX101814.2	6	71.79	33.5
OX101815.2	7	71.66	34.0
OX101816.2	8	69.73	33.0
OX101817.2	9	69.62	33.5
OX101818.2	10	69.29	35.0
OX101819.2	11	66.75	33.5
OX101820.2	12	63.86	34.0
OX101822.2	14	62.92	33.5
OX101821.2	13	62.58	33.5
OX101823.2	15	61.04	34.0
OX101824.2	16	58.48	33.0
OX101825.2	17	56.8	34.0
OX101826.2	18	53.51	33.0
OX101827.2	19	48.61	34.0
OX101828.2	20	45.71	33.5
OX101829.2	21	20.53	31.5
OX101809.2	X	79.6	34.5
OX101830.2	MT	0.02	24.0

The estimated Quality Value (QV) of the final assembly is 57.8 with *k*-mer completeness of 99.99%, and the assembly has a BUSCO v5.3.2 completeness of 99.3% (single = 98.4%, duplicated = 0.9%), using the endopterygota_odb10 reference set ( *n* = 2,124).

Metadata for specimens, spectral estimates, sequencing runs, contaminants and pre-curation assembly statistics can be found at https://links.tol.sanger.ac.uk/species/1587174.

## Genome annotation report

The *Chrysolina oricalcia* genome assembly (GCA_944452925.2) was annotated using the Ensembl rapid annotation pipeline ( Table 1; https://rapid.ensembl.org/Chrysolina_oricalcia_GCA_944452925.1/Info/Index). The resulting annotation includes 36,271 transcribed mRNAs from 35,990 protein-coding genes.

## Methods

### Sample acquisition and nucleic acid extraction

A single specimen of *Chrysolina oricalcia* (specimen ID NHMUK014111041, ToLID icChrOric1) was collected on 2020-06-02 from a woodland edge at Wigmore Park (51.88, –0.37), Percival Way, Wigmore, Luton, England, by Olga Sivell, Natural History Museum, London. The morphological identification was provided by Duncan Sivell, Natural History Museum, London, based on Duff (2016). The sample was snap-frozen using dry ice and stored in a CoolRack.

DNA was extracted at the Tree of Life laboratory, Wellcome Sanger Institute (WSI). The icChrOric1 sample was weighed and dissected on dry ice with tissue set aside for Hi-C sequencing. Tissue from the abdomen was cryogenically disrupted to a fine powder using a Covaris cryoPREP Automated Dry Pulveriser, receiving multiple impacts. High molecular weight (HMW) DNA was extracted using the Qiagen MagAttract HMW DNA extraction kit. Low molecular weight DNA was removed from a 20 ng aliquot of extracted DNA using the 0.8X AMpure XP purification kit prior to 10X Chromium sequencing; a minimum of 50 ng DNA was submitted for 10X sequencing. HMW DNA was sheared into an average fragment size of 12–20 kb in a Megaruptor 3 system with speed setting 30. Sheared DNA was purified by solid-phase reversible immobilisation using AMPure PB beads with a 1.8X ratio of beads to sample to remove the shorter fragments and concentrate the DNA sample. The concentration of the sheared and purified DNA was assessed using a Nanodrop spectrophotometer and Qubit Fluorometer and Qubit dsDNA High Sensitivity Assay kit. Fragment size distribution was evaluated by running the sample on the FemtoPulse system.

### Sequencing

Pacific Biosciences HiFi circular consensus and 10X Genomics read cloud DNA sequencing libraries were constructed according to the manufacturers’ instructions. DNA sequencing was performed by the Scientific Operations core at the WSI on Pacific Biosciences SEQUEL II (HiFi) and HiSeq X Ten (10X) instruments. Hi-C data were also generated from remaining abdomen tissue of icChrOric1 using the Arima2 kit and sequenced on the Illumina NovaSeq 6000 instrument.

### Genome assembly, curation and evaluation

Assembly was carried out with Hifiasm ( Cheng *et al.*, 2021) and haplotypic duplication was identified and removed with purge_dups ( Guan *et al.*, 2020). One round of polishing was performed by aligning 10X Genomics read data to the assembly with Long Ranger ALIGN, calling variants with FreeBayes ( Garrison & Marth, 2012). The assembly was then scaffolded with Hi-C data ( Rao *et al.*, 2014) using YaHS ( Zhou *et al.*, 2023). The assembly was checked for contamination and corrected using the gEVAL system ( Chow *et al.*, 2016) as described previously ( Howe *et al.*, 2021). Manual curation was performed using gEVAL, HiGlass ( Kerpedjiev *et al.*, 2018) and Pretext ( Harry, 2022). The mitochondrial genome was assembled using MitoHiFi ( Uliano-Silva *et al.*, 2023), which runs MitoFinder ( Allio *et al.*, 2020) or MITOS ( Bernt *et al.*, 2013) and uses these annotations to select the final mitochondrial contig and to ensure the general quality of the sequence.

A Hi-C map for the final assembly was produced using bwa-mem2 ( Vasimuddin *et al.*, 2019) in the Cooler file format ( Abdennur & Mirny, 2020). To assess the assembly metrics, the *k*-mer completeness and QV consensus quality values were calculated in Merqury ( Rhie *et al.*, 2020). This work was done using Nextflow ( Di Tommaso *et al.*, 2017) DSL2 pipelines “sanger-tol/readmapping” ( Surana *et al.*, 2023a) and “sanger-tol/genomenote” ( Surana *et al.*, 2023b). The genome was analysed within the BlobToolKit environment ( Challis *et al.*, 2020) and BUSCO scores ( Manni *et al.*, 2021; Simão *et al.*, 2015) were calculated.

Table 3 contains a list of relevant software tool versions and sources.

**Table 3.** Software tools: versions and sources.

Software tool	Version	Source
BlobToolKit	4.1.7	https://github.com/blobtoolkit/blobtoolkit
BUSCO	5.3.2	https://gitlab.com/ezlab/busco
FreeBayes	1.3.1-17- gaa2ace8	https://github.com/freebayes/freebayes
gEVAL	N/A	https://geval.org.uk/
Hifiasm	0.12	https://github.com/chhylp123/hifiasm
HiGlass	1.11.6	https://github.com/higlass/higlass
Long Ranger ALIGN	2.2.2	https://support.10xgenomics.com/genome-exome/software/pipelines/latest/advanced/other-pipelines
Merqury	MerquryFK	https://github.com/thegenemyers/MERQURY.FK
MitoHiFi	2	https://github.com/marcelauliano/MitoHiFi
PretextView	0.2	https://github.com/wtsi-hpag/PretextView
purge_dups	1.2.3	https://github.com/dfguan/purge_dups
sanger-tol/ genomenote	v1.0	https://github.com/sanger-tol/genomenote
sanger-tol/ readmapping	1.1.0	https://github.com/sanger-tol/readmapping/tree/1.1.0
YaHS	yahs- 1.1.91eebc2	https://github.com/c-zhou/yahs

### Genome annotation

The BRAKER2 pipeline ( Brůna *et al.*, 2021) was used in the default protein mode to generate annotation for the *Chrysolina oricalcia* assembly (GCA_944452925.1) in Ensembl Rapid Release.

### Wellcome Sanger Institute – Legal and Governance

The materials that have contributed to this genome note have been supplied by a Darwin Tree of Life Partner. The submission of materials by a Darwin Tree of Life Partner is subject to the **‘Darwin Tree of Life Project Sampling Code of Practice’**, which can be found in full on the Darwin Tree of Life website here. By agreeing with and signing up to the Sampling Code of Practice, the Darwin Tree of Life Partner agrees they will meet the legal and ethical requirements and standards set out within this document in respect of all samples acquired for, and supplied to, the Darwin Tree of Life Project. 

Further, the Wellcome Sanger Institute employs a process whereby due diligence is carried out proportionate to the nature of the materials themselves, and the circumstances under which they have been/are to be collected and provided for use. The purpose of this is to address and mitigate any potential legal and/or ethical implications of receipt and use of the materials as part of the research project, and to ensure that in doing so we align with best practice wherever possible. The overarching areas of consideration are:

• Ethical review of provenance and sourcing of the material

• Legality of collection, transfer and use (national and international) 

Each transfer of samples is further undertaken according to a Research Collaboration Agreement or Material Transfer Agreement entered into by the Darwin Tree of Life Partner, Genome Research Limited (operating as the Wellcome Sanger Institute), and in some circumstances other Darwin Tree of Life collaborators.

## Data Availability

European Nucleotide Archive: *Chrysolina oricalcia*. Accession number PRJEB52653; https://identifiers.org/ena.embl/PRJEB52653. ( Wellcome Sanger Institute, 2022) The genome sequence is released openly for reuse. The *Chrysolina oricalcia* genome sequencing initiative is part of the Darwin Tree of Life (DToL) project. All raw sequence data and the assembly have been deposited in INSDC databases. Raw data and assembly accession identifiers are reported in Table 1.

## Author information

Members of the Natural History Museum Genome Acquisition Lab are listed here: https://doi.org/10.5281/zenodo.4790042.

Members of the Darwin Tree of Life Barcoding collective are listed here: https://doi.org/10.5281/zenodo.4893703.

Members of the Wellcome Sanger Institute Tree of Life programme are listed here: https://doi.org/10.5281/zenodo.4783585.

Members of Wellcome Sanger Institute Scientific Operations: DNA Pipelines collective are listed here: https://doi.org/10.5281/zenodo.4790455.

Members of the Tree of Life Core Informatics collective are listed here: https://doi.org/10.5281/zenodo.5013541.

Members of the Darwin Tree of Life Consortium are listed here: https://doi.org/10.5281/zenodo.4783558.
