# Supplementary material for: The genome sequence of the Cow Parsley Leaf Beetle, Chrysolina oricalcia (O.F. Müller, 1776)
Source: Wellcome Open Res. 2024 Jul 18;8:400. Originally published 2023 Sep 11. [Version 2] doi: 10.12688/wellcomeopenres.19985.2 (PMC11347920; doi:10.12688/wellcomeopenres.19985.2)
Supplement: Peer review history

# Reviewer report: Reviewer response for version 2

**DOI:** 10.21956/wellcomeopenres.25054.r105885

**Published:** 2024-12-09

**Authors:** Lijie Zhang, Lijie Jin

**Affiliations:** 1 Science and Technical Research Center of China Customs, Beijing, China

1. Abstract, last paragraph in Introduction, first paragraph in Methods: Please indicate whether it is male or female. 

 2. The second paragraph in Introduction: What are the other obvious identification characteristics besides information such as color and body length?

Are sufficient details of methods and materials provided to allow replication by others?

Yes

Is the rationale for creating the dataset(s) clearly described?

Yes

Are the datasets clearly presented in a useable and accessible format?

Yes

Are the protocols appropriate and is the work technically sound?

Yes

Reviewer Expertise:

coleoptera diversity and distribution

We confirm that we have read this submission and believe that we have an appropriate level of expertise to confirm that it is of an acceptable scientific standard, however we have significant reservations, as outlined above.

# Reviewer report: Reviewer response for version 2

**DOI:** 10.21956/wellcomeopenres.25054.r105891

**Published:** 2024-11-05

**Authors:** Manuel Barrios-Izas

**Affiliations:** 1 University of San Carlos of Guatemala, Zacapa, Guatemala

There is a typo "(Figure 1b))" in line 1 of Genome sequence report section, change by "(Figure 1b)".

**General Comments:** The document is well-written and clear on several important aspects:

- **Taxonomic and geographical information**: These details are comprehensive and easy to understand.
- **Diagnostic characters**: They are well described.
- **DNA extraction and isolation methods**: The methods are clearly explained and reliable.
- **Bioinformatic analyses**: These are well-structured and thoroughly explained.
- **Genome assembly quality**: The assembly is of high quality, reflecting the authors' meticulous work.
- **Data availability**: The data are accessible, which is valuable for reproducibility and future research.

Overall, the authors have done an excellent job in presenting and documenting the genome of the studied species.

Are sufficient details of methods and materials provided to allow replication by others?

Yes

Is the rationale for creating the dataset(s) clearly described?

Yes

Are the datasets clearly presented in a useable and accessible format?

Yes

Are the protocols appropriate and is the work technically sound?

Yes

Reviewer Expertise:

Entomology, Bioinformatics, Systematics

I confirm that I have read this submission and believe that I have an appropriate level of expertise to confirm that it is of an acceptable scientific standard.

# Reviewer report: Reviewer response for version 2

**DOI:** 10.21956/wellcomeopenres.25054.r90189

**Published:** 2024-09-05

**Authors:** Fahad Alqahtani

**Affiliations:** 1 King Abdulaziz City for Science and Technology, Riyadh, Saudi Arabia

I am happy with the manuscript in its current form.

Are sufficient details of methods and materials provided to allow replication by others?

Yes

Is the rationale for creating the dataset(s) clearly described?

Yes

Are the datasets clearly presented in a useable and accessible format?

Yes

Are the protocols appropriate and is the work technically sound?

Yes

Reviewer Expertise:

Bioinformatics

I confirm that I have read this submission and believe that I have an appropriate level of expertise to confirm that it is of an acceptable scientific standard.

# Reviewer report: Reviewer response for version 1

**DOI:** 10.21956/wellcomeopenres.22131.r74802

**Published:** 2024-08-26

**Authors:** Themistoklis Giannoulis

**Affiliations:** 1 Laboratory of Genetics, Faculty of Animal Science, University of Thessaly, Larissa, Greece

First of all, I would like to mention that I am a huge fan of the effort "One genome for each species"; this manuscript is towards this goal and provides a genome for the *C. oricalcia*. The results are impressive, since the authors combined two sequencing methods and achieved a chromosome level assembly for the 99% of the sequences. The availability of such data is really useful, especially in the field of molecular ecology and conservation genetics. The manuscript is well written, the methods are clearly described and the results as well.

Are sufficient details of methods and materials provided to allow replication by others?

Yes

Is the rationale for creating the dataset(s) clearly described?

Yes

Are the datasets clearly presented in a useable and accessible format?

Yes

Are the protocols appropriate and is the work technically sound?

Yes

Reviewer Expertise:

Animal genetics and genomics

I confirm that I have read this submission and believe that I have an appropriate level of expertise to confirm that it is of an acceptable scientific standard.

# Reviewer report: Reviewer response for version 1

**DOI:** 10.21956/wellcomeopenres.22131.r74810

**Published:** 2024-03-04

**Authors:** Richard Adams

**Affiliations:** 1 University of Arkansas Fayetteville, Fayetteville, Arkansas, USA

Thank you for consideration of my review of “The genome sequence of the Cow Parsley Leaf Beetle, Chrysolina oricalcia (O.F. Müller, 1776)”, which describes new genomic data, assembly, annotation, and associated resources for an interesting Chrysomelid beetle inhabiting the British Isles. The authors generated a battery of next-gen sequencing data for *C. oricalcia*, including HiFi, 10x Genomics, and Hi-C data. They assembled 82 contigs spanning ~79Mb of the genome (21 autosomes + X chromosome), and applied the Ensembl rapid pipeline to annotate the assembly.  

 Overall, your Data Note is nicely and succinctly written, and also timely, as I am certain it will be appreciated in the entomological community and more broadly across evolutionary genomics. The megadiversity of Coleoptera is vastly under-sampled and under-appreciated, and thus, these new genomic resources will contribute to a growing understanding of genome diversity and evolution in this clade. I have a couple of suggestions here that deigned to improve the clarity, impact, and accessibility of your Data Note. Please don’t hesitate to reach out with any questions if helpful.

 A couple of suggestions:

1. The Genome Sequence Report section could benefit from a few sentences describing the quality of the genome annotation. For example, The Rapid pipeline suggested ~36k distinct protein coding genes. Is it possible to elaborate here? This seems a little on the high-side, but BUSCO suggested only 0.8% are duplicated. For the sake of understanding assembly quality, it would be helpful to at least qualitatively compare your assembly to other recently assembled beetle genomes. For example, is this number comparative to a small sampling of other insect genomes in similar quality? A brief sentence or two would be helpful to place your Data Note/Annotation quality in perspective. Data Notes discourage further analyses or conclusions, so I don’t suggest any formal analyses here, just a little insight into the quality of the annotation, which many would appreciate.
2. Increasing the size and font of your figures would help interpretability. The in-text rendering is difficult to see the small points, etc. (e.g., Fig. 3)
3. Figure 2 could benefit from some clarification in the caption and/or figure itself. For example, color description, bp resolution, and would it be possible to add chromosome labels?
4. Would it be possible to provide a (even course-scale) breakdown of the repeat element composition (e.g., RepeatMasker suite) of the *C. oricalcia *genome?
5. As I side note, I really appreciate the inclusion of interactive figures (very nice!)

Are sufficient details of methods and materials provided to allow replication by others?

Yes

Is the rationale for creating the dataset(s) clearly described?

Yes

Are the datasets clearly presented in a useable and accessible format?

Yes

Are the protocols appropriate and is the work technically sound?

Yes

Reviewer Expertise:

Bioinformatics, Computational Genomics, Phylogenetics

I confirm that I have read this submission and believe that I have an appropriate level of expertise to confirm that it is of an acceptable scientific standard.

# Reviewer report: Reviewer response for version 1

**DOI:** 10.21956/wellcomeopenres.22131.r74809

**Published:** 2024-02-26

**Authors:** Fahad Alqahtani

**Affiliations:** 1 King Abdulaziz City for Science and Technology, Riyadh, Saudi Arabia

The authors stated that they successfully reconstructed a high-completeness genome assembly at a chromosomal level and high-quality annotation for a male Cow Parsley Leaf Beetle Chrysolina oricalcia (O.F. Müller, 1776). The assembly was performed using three sequencing technologies: PacificBiosciences SEQUEL II, 10X Genomics Illumina, and Hi-C Illumina. The genome size is approximately 1,423.4 Mb. The completeness of genome assembly was determined using BUSCO analysis which is a 99.3% of common genes were completely present. However, there are minor comments that need to be addressed:

- I observed that the authors stated the sample was collected from male Chrysolina oricalcia. However, according to the NCBI BioSample database, the sample's sex is listed as "NOT COLLECTED." It would be better for the BioSample information to be updated to reflect the male sex of the sample.
- Additionally, the photo of Chrysolina oricalcia in Figure 1-b should be improved for better visualization.
- Regarding the mitochondrial genome annotation, the use of the Mitofinder tool, which employs closely related species to guide the annotation. the authors should mention of the related species is used. Furthermore, since the authors utilized both MITOS and MitoFinder for annotation, a detailed explanation of how these tools were used to select the final mitochondrial contig would increase the reader's understanding.
- In the Genome Assembly section, the tool "Pretext" should be changed to be "PretextView."
- Lastly, the paper has a confusion about versions: when showing chromosomes, it mentions the second version, but then goes back to the first version when talking about genome annotation. Explaining this mix-up would help keep the paper clear and accurate.

Are sufficient details of methods and materials provided to allow replication by others?

Yes

Is the rationale for creating the dataset(s) clearly described?

Yes

Are the datasets clearly presented in a useable and accessible format?

Yes

Are the protocols appropriate and is the work technically sound?

Yes

Reviewer Expertise:

Bioinformatics

I confirm that I have read this submission and believe that I have an appropriate level of expertise to confirm that it is of an acceptable scientific standard, however I have significant reservations, as outlined above.
